# 2-Methyl­pyridinium 5-(2,4-dinitro­phen­yl)-1,3-dimethyl­barbiturate

**DOI:** 10.1107/S1600536812009440

**Published:** 2012-03-14

**Authors:** Gunaseelan Sridevi, Doraisamyraja Kalaivani

**Affiliations:** a PG and Research Department of Chemistry, Seethalakshmi Ramaswami College, Tiruchirappalli 620 002, Tamil Nadu, India

## Abstract

In the title mol­ecular salt [systematic name: 2-methyl­pyridinium 5-(2,4-dinitro­phen­yl)-1,3-dimethyl-2,6-dioxo-1,2,3,6-tetra­hydro­pyrimidin-4-olate], C_6_H_8_N^+^·C_12_H_9_N_4_O_7_^−^, the cation and anion are linked a through strong N—H⋯O hydrogen bond. In the crystal, C—H⋯O inter­actions link the ions, generating a chain along [010].

## Related literature

For the biological properties of mol­ecules containing pyridine and pyrimidine units, see: Terekhova & Scriba (2007 ▶); Comins *et al.* (2008 ▶); Hueso *et al.* (2003 ▶); Jain *et al.* (2006 ▶). For the structures of barbiturates similar to the title compound, see: Kalaivani & Malarvizhi (2009 ▶); Kalaivani & Buvaneswari (2010 ▶); Buvaneswari & Kalaivani (2011 ▶).
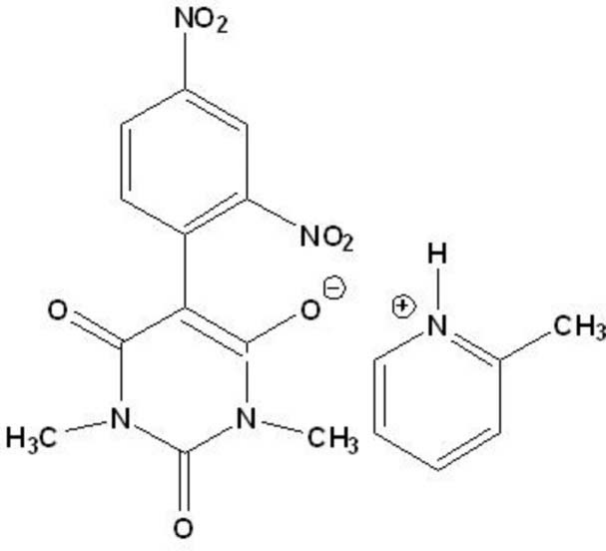


## Experimental

### 

#### Crystal data

C_6_H_8_N^+^·C_12_H_9_N_4_O_7_^−^
*M* *_r_* = 415.37
Monoclinic, 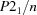

*a* = 12.8242 (8) Å
*b* = 7.0696 (5) Å
*c* = 21.5409 (14) Å
β = 101.029 (2)°
*V* = 1916.9 (2) Å^3^
*Z* = 4
Mo *K*α radiation
μ = 0.11 mm^−1^
*T* = 293 K
0.30 × 0.25 × 0.15 mm

#### Data collection

Bruker Kappa APEXII CCD diffractometer
Absorption correction: multi-scan (*SADABS*; Bruker, 2004 ▶) *T*_min_ = 0.917, *T*_max_ = 0.983
3527 measured reflections
3527 independent reflections
2790 reflections with *I* > 2σ(*I*)

#### Refinement

*R*[*F* ^2^ > 2σ(*F* ^2^)] = 0.036
*wR*(*F* ^2^) = 0.099
*S* = 1.04
3527 reflections
275 parameters
H-atom parameters constrained
Δρ_max_ = 0.15 e Å^−3^
Δρ_min_ = −0.17 e Å^−3^

### 

Data collection: *APEX2* (Bruker, 2004 ▶); cell refinement: *SAINT* (Bruker, 2004 ▶); data reduction: *SAINT*; program(s) used to solve structure: *SIR92* (Altomare *et al.*, 1993 ▶); program(s) used to refine structure: *SHELXL97* (Sheldrick, 2008 ▶); molecular graphics: *ORTEP-3* (Farrugia, 1997 ▶) and *Mercury* (Macrae *et al.*, 2008 ▶); software used to prepare material for publication: *PLATON* (Spek, 2009 ▶).

## Supplementary Material

Crystal structure: contains datablock(s) global, I. DOI: 10.1107/S1600536812009440/bv2197sup1.cif

Structure factors: contains datablock(s) I. DOI: 10.1107/S1600536812009440/bv2197Isup2.hkl

Supplementary material file. DOI: 10.1107/S1600536812009440/bv2197Isup3.cml

Additional supplementary materials:  crystallographic information; 3D view; checkCIF report

## Figures and Tables

**Table 1:** Hydrogen-bond geometry (Å, °)

*D*—H⋯*A*	*D*—H	H⋯*A*	*D*⋯*A*	*D*—H⋯*A*
N5—H5*A*⋯O3	0.86	1.82	2.6645 (16)	168
C13—H13*B*⋯O5^i^	0.96	2.42	3.340 (2)	161
C13—H13*C*⋯O1^ii^	0.96	2.42	3.160 (2)	134
C15—H15⋯O2^iii^	0.93	2.29	3.021 (2)	135
C16—H16⋯O6^iv^	0.93	2.58	3.323 (2)	138
C17—H17⋯O1^v^	0.93	2.52	3.303 (2)	143

Symmetry codes: (i) 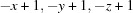; (ii) 
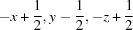
; (iii) 
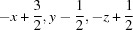
; (iv) 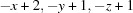; (v) 
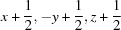
.

**Fig. 1.**
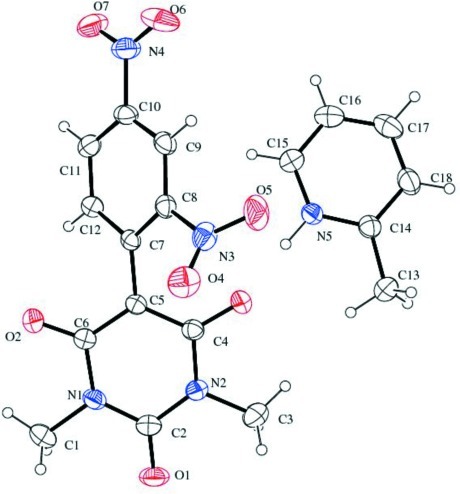
The asymmetric unit of title molecule showing 30% probability displacement ellipsoids.

**Fig. 2.**
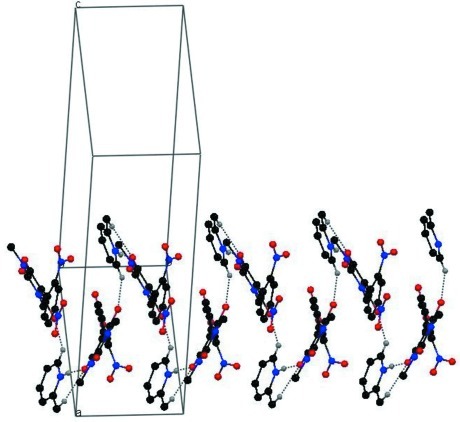
Packing view of title molecule showing the chains in the [010] direction.

## supplementary crystallographic information

### Comment

Many molecules containing the pyridine moiety exhibit notable biological activity (Terekhova & Scriba, 2007; Comins *et al.*, 2008). Molecules with pyrimidine ring residues are also biologically active (Hueso *et al.*, 2003; Jain *et al.*, 2006). The title molecular salt comprises of both pyrimidine (barbiturate moiety) and pyridine (2-methyl pyridinium) moieties and hence expected to have significant biological activity. Good crystallinity of the title compound prompted us to undertake single-crystal X-ray studies. The bond lengths and bond angles of the barbiturate residue of the molecular salt reported in the present article are compatible with those of related barbiturates synthesized in our laboratory earlier (Kalaivani & Malarvizhi, 2009; Kalaivani & Buvaneswari, 2010; Buvaneswari & Kalaivani, 2011). The structure of the molecular salt of the present work is shown in scheme 1. The *ORTEP* view showing 30% probability displacement ellipsoids is indicated in Fig.1. In the title molecule, the one dimensional zigzag chains which run along [010] direction are linked through C13—H13···O1, C17—H17···O1, C16—H16···O6, C13—H13···O3 and C15—H15···O2 weak interactions thus generating a three dimensional network and hence constituting the molecular packing of the crystal (Fig. 2). The 2,4-dinitrophenyl ring and 1,3-dimethylbarbiturate ring of the title molecule are not perfectly planar and the dihedral angle observed between them is 44.54 (2)degree.

### Experimental

1-Chloro-2,4-dinitrobenzene(2.02 g,0.01 mol) was dissolved in 20 ml of ethanol. 1,3-Dimethylbarbituric acid (1.56 g,0.01 mol) was also dissolved in 15 ml of ethanol. These two solutions were mixed and to this mixture a five fold excess of 2-methylpyridine (4.65 g, 0.05 mol) was added and the resulting blood red coloured solution was shaken well for about three hours and kept as such at 25 /%C. Dark shiny maroon red coloured crystals were deposited from the solution after seventy two hours. The crystals were filtered and washed with 30 ml of ether. The crystals were powdered well and washed with 40 ml of ether to remove the unreacted reactants and finally with 10 ml of ethanol. The pure powder was recrystallized from hot ethanol (Yield: 75%; m.p.; 451 K). The crystals for X-ray analysis were obtained by slow evaporation of ethanol at room temperature.

### Refinement

All of the H atoms were placed in their calculated positions and then refined using the riding model with C—H lengths of 0.93Å (CH), 0.96Å (CH_2_) or 0.98Å (CH_3_) and an N—H distance of 0.86 Å. The isotropic displacement parameters for these atoms were set to 1.2 (CH, and NH) or 1.50 times *U*_eq_ (CH_3_) of the parent atom.

### Crystal data

**Table:**

C_6_H_8_N^+^·C_12_H_9_N_4_O_7_^−^	*F*(000) = 864
*M**_r_* = 415.37	*D*_x_ = 1.439 Mg m^−^^3^
Monoclinic, *P*2_1_/*n*	Mo *K*α radiation, λ = 0.71073 Å
Hall symbol: -P 2yn	Cell parameters from 6197 reflections
*a* = 12.8242 (8) Å	θ = 2.9–25.3°
*b* = 7.0696 (5) Å	µ = 0.11 mm^−^^1^
*c* = 21.5409 (14) Å	*T* = 293 K
β = 101.029 (2)°	Block, red
*V* = 1916.9 (2) Å^3^	0.30 × 0.25 × 0.15 mm
*Z* = 4	

### Data collection

**Table:**

Bruker Kappa APEXII CCD diffractometer	3527 independent reflections
Radiation source: fine-focus sealed tube	2790 reflections with *I* > 2σ(*I*)
Graphite monochromator	*R*_int_ = 0.000
ω and φ scan	θ_max_ = 25.4°, θ_min_ = 3.0°
Absorption correction: multi-scan (*SADABS*; Bruker, 2004)	*h* = −15→15
*T*_min_ = 0.917, *T*_max_ = 0.983	*k* = 0→8
3527 measured reflections	*l* = 0→25

### Refinement

**Table:**

Refinement on *F*^2^	Primary atom site location: structure-invariant direct methods
Least-squares matrix: full	Secondary atom site location: difference Fourier map
*R*[*F*^2^ > 2σ(*F*^2^)] = 0.036	Hydrogen site location: inferred from neighbouring sites
*wR*(*F*^2^) = 0.099	H-atom parameters constrained
*S* = 1.04	*w* = 1/[σ^2^(*F*_o_^2^) + (0.045*P*)^2^ + 0.4365*P*] where *P* = (*F*_o_^2^ + 2*F*_c_^2^)/3
3527 reflections	(Δ/σ)_max_ < 0.001
275 parameters	Δρ_max_ = 0.15 e Å^−^^3^
0 restraints	Δρ_min_ = −0.17 e Å^−^^3^

### Special details

**Table:**

Geometry. All esds (except the esd in the dihedral angle between two l.s. planes) are estimated using the full covariance matrix. The cell esds are taken into account individually in the estimation of esds in distances, angles and torsion angles; correlations between esds in cell parameters are only used when they are defined by crystal symmetry. An approximate (isotropic) treatment of cell esds is used for estimating esds involving l.s. planes.
Refinement. Refinement of F^2^ against ALL reflections. The weighted R-factor wR and goodness of fit S are based on F^2^, conventional R-factors R are based on F, with F set to zero for negative F^2^. The threshold expression of F^2^ > 2sigma(F^2^) is used only for calculating R-factors(gt) etc. and is not relevant to the choice of reflections for refinement. R-factors based on F^2^ are statistically about twice as large as those based on F, and R- factors based on ALL data will be even larger.

### Fractional atomic coordinates and isotropic or equivalent isotropic displacement parameters (Å^2^)

**Table:**

	*x*	*y*	*z*	*U*_iso_*/*U*_eq_	Occ. (<1)
O1	0.25790 (8)	0.59806 (19)	0.13771 (6)	0.0652 (4)	
O2	0.58313 (8)	0.84817 (17)	0.13910 (5)	0.0521 (3)	
O3	0.49962 (8)	0.55652 (17)	0.31960 (5)	0.0550 (3)	
O4	0.53244 (10)	0.9752 (2)	0.33842 (6)	0.0694 (4)	
O5	0.60991 (12)	0.8263 (2)	0.42199 (6)	0.0831 (4)	
O6	0.98964 (12)	0.8224 (3)	0.43012 (8)	0.1054 (6)	
O7	1.04967 (11)	0.7451 (2)	0.34758 (8)	0.0851 (5)	
N1	0.41979 (10)	0.72838 (19)	0.13974 (6)	0.0457 (3)	
N2	0.37859 (9)	0.58307 (18)	0.22914 (6)	0.0453 (3)	
N3	0.60166 (12)	0.8717 (2)	0.36639 (6)	0.0552 (4)	
N4	0.97702 (13)	0.7814 (2)	0.37450 (9)	0.0678 (5)	
C1	0.38684 (15)	0.7872 (3)	0.07370 (8)	0.0711 (6)	
H1A	0.4128	0.9125	0.0685	0.107*	0.62 (3)
H1B	0.3107	0.7867	0.0625	0.107*	0.62 (3)
H1C	0.4154	0.7011	0.0468	0.107*	0.62 (3)
H1C1	0.4484	0.8199	0.0567	0.107*	0.38 (3)
H1C2	0.3408	0.8951	0.0715	0.107*	0.38 (3)
H1C3	0.3497	0.6853	0.0496	0.107*	0.38 (3)
C2	0.34662 (11)	0.6346 (2)	0.16695 (7)	0.0461 (4)	
C3	0.30525 (14)	0.4711 (3)	0.25827 (10)	0.0714 (6)	
H3A	0.2900	0.5371	0.2945	0.107*	
H3B	0.3370	0.3510	0.2713	0.107*	
H3C	0.2405	0.4516	0.2282	0.107*	
C4	0.48022 (11)	0.6176 (2)	0.26412 (7)	0.0404 (3)	
C5	0.55251 (10)	0.71463 (19)	0.23462 (6)	0.0363 (3)	
C6	0.52461 (11)	0.7686 (2)	0.17015 (7)	0.0390 (3)	
C7	0.65998 (11)	0.74854 (18)	0.26967 (6)	0.0351 (3)	
C8	0.68428 (11)	0.8054 (2)	0.33312 (7)	0.0412 (3)	
C9	0.78652 (12)	0.8162 (2)	0.36788 (7)	0.0476 (4)	
H9	0.7991	0.8498	0.4104	0.057*	
C10	0.86841 (12)	0.7759 (2)	0.33778 (8)	0.0472 (4)	
C11	0.85129 (12)	0.7277 (2)	0.27469 (8)	0.0466 (4)	
H11	0.9082	0.7047	0.2547	0.056*	
C12	0.74849 (11)	0.7141 (2)	0.24194 (7)	0.0405 (3)	
H12	0.7372	0.6805	0.1994	0.049*	
N5	0.61757 (9)	0.36331 (18)	0.41383 (6)	0.0434 (3)	
H5A	0.5829	0.4153	0.3800	0.052*	
C13	0.44507 (13)	0.3047 (3)	0.43878 (8)	0.0616 (5)	
H13A	0.4246	0.4261	0.4205	0.092*	
H13B	0.4158	0.2883	0.4762	0.092*	
H13C	0.4187	0.2072	0.4089	0.092*	
C14	0.56244 (12)	0.2933 (2)	0.45537 (7)	0.0447 (4)	
C15	0.72358 (12)	0.3570 (2)	0.42194 (8)	0.0530 (4)	
H15	0.7579	0.4067	0.3913	0.064*	
C16	0.78080 (14)	0.2779 (3)	0.47504 (9)	0.0638 (5)	
H16	0.8545	0.2717	0.4811	0.077*	
C17	0.72821 (16)	0.2074 (3)	0.51962 (9)	0.0686 (5)	
H17	0.7664	0.1546	0.5566	0.082*	
C18	0.61948 (15)	0.2143 (3)	0.50993 (8)	0.0602 (5)	
H18	0.5841	0.1655	0.5403	0.072*	

### Atomic displacement parameters (Å^2^)

**Table:**

	*U*^11^	*U*^22^	*U*^33^	*U*^12^	*U*^13^	*U*^23^
O1	0.0384 (6)	0.0833 (9)	0.0665 (7)	−0.0017 (6)	−0.0081 (5)	−0.0018 (7)
O2	0.0521 (6)	0.0640 (7)	0.0406 (6)	−0.0043 (5)	0.0101 (5)	0.0080 (5)
O3	0.0508 (6)	0.0660 (8)	0.0458 (6)	−0.0074 (5)	0.0030 (5)	0.0188 (5)
O4	0.0682 (8)	0.0715 (9)	0.0722 (8)	0.0120 (7)	0.0225 (7)	−0.0144 (7)
O5	0.1004 (10)	0.1142 (12)	0.0405 (7)	−0.0185 (9)	0.0278 (7)	−0.0079 (7)
O6	0.0762 (10)	0.1480 (16)	0.0739 (10)	−0.0106 (10)	−0.0311 (8)	−0.0053 (10)
O7	0.0414 (7)	0.0957 (11)	0.1117 (12)	−0.0104 (7)	−0.0020 (8)	0.0226 (9)
N1	0.0428 (7)	0.0528 (8)	0.0379 (7)	0.0017 (6)	−0.0018 (5)	0.0019 (6)
N2	0.0368 (6)	0.0482 (8)	0.0497 (7)	−0.0033 (5)	0.0050 (5)	0.0045 (6)
N3	0.0637 (9)	0.0614 (9)	0.0440 (8)	−0.0126 (8)	0.0187 (7)	−0.0133 (7)
N4	0.0493 (9)	0.0633 (10)	0.0807 (12)	−0.0116 (7)	−0.0128 (8)	0.0157 (9)
C1	0.0662 (11)	0.0939 (15)	0.0451 (10)	−0.0040 (10)	−0.0097 (8)	0.0135 (10)
C2	0.0386 (8)	0.0465 (9)	0.0505 (9)	0.0043 (7)	0.0021 (7)	−0.0032 (7)
C3	0.0498 (10)	0.0804 (14)	0.0828 (13)	−0.0159 (9)	0.0093 (9)	0.0196 (11)
C4	0.0389 (7)	0.0388 (8)	0.0423 (8)	0.0018 (6)	0.0048 (6)	0.0017 (6)
C5	0.0374 (7)	0.0344 (7)	0.0360 (7)	0.0018 (6)	0.0047 (6)	−0.0009 (6)
C6	0.0398 (7)	0.0373 (8)	0.0393 (8)	0.0034 (6)	0.0059 (6)	−0.0030 (6)
C7	0.0399 (7)	0.0289 (7)	0.0356 (7)	−0.0002 (6)	0.0050 (6)	0.0019 (5)
C8	0.0468 (8)	0.0383 (8)	0.0384 (8)	−0.0052 (6)	0.0084 (6)	0.0009 (6)
C9	0.0556 (9)	0.0452 (9)	0.0378 (8)	−0.0107 (7)	−0.0018 (7)	0.0024 (7)
C10	0.0413 (8)	0.0397 (8)	0.0541 (9)	−0.0072 (7)	−0.0073 (7)	0.0089 (7)
C11	0.0395 (8)	0.0399 (8)	0.0607 (10)	0.0014 (6)	0.0102 (7)	0.0050 (7)
C12	0.0425 (7)	0.0374 (8)	0.0411 (8)	0.0030 (6)	0.0062 (6)	−0.0019 (6)
N5	0.0440 (7)	0.0465 (7)	0.0380 (6)	−0.0003 (6)	0.0035 (5)	0.0016 (6)
C13	0.0480 (9)	0.0805 (13)	0.0576 (10)	−0.0059 (9)	0.0132 (8)	0.0028 (9)
C14	0.0498 (8)	0.0434 (9)	0.0402 (8)	−0.0049 (7)	0.0070 (7)	−0.0030 (7)
C15	0.0449 (9)	0.0567 (10)	0.0574 (10)	−0.0032 (7)	0.0097 (7)	−0.0012 (8)
C16	0.0472 (9)	0.0676 (12)	0.0707 (12)	0.0041 (8)	−0.0040 (9)	−0.0013 (10)
C17	0.0714 (12)	0.0662 (12)	0.0584 (11)	0.0080 (10)	−0.0121 (9)	0.0101 (9)
C18	0.0725 (12)	0.0598 (11)	0.0466 (9)	−0.0047 (9)	0.0071 (8)	0.0110 (8)

### Geometric parameters (Å, º)

**Table:**

O1—C2	1.2181 (17)	C5—C7	1.4591 (18)
O2—C6	1.2325 (17)	C7—C12	1.401 (2)
O3—C4	1.2501 (17)	C7—C8	1.4014 (19)
O4—N3	1.2174 (19)	C8—C9	1.382 (2)
O5—N3	1.2248 (18)	C9—C10	1.366 (2)
O6—N4	1.213 (2)	C9—H9	0.9300
O7—N4	1.215 (2)	C10—C11	1.377 (2)
N1—C2	1.369 (2)	C11—C12	1.374 (2)
N1—C6	1.4071 (18)	C11—H11	0.9300
N1—C1	1.465 (2)	C12—H12	0.9300
N2—C2	1.3728 (19)	N5—C14	1.3370 (19)
N2—C4	1.3966 (18)	N5—C15	1.3379 (19)
N2—C3	1.460 (2)	N5—H5A	0.8600
N3—C8	1.465 (2)	C13—C14	1.481 (2)
N4—C10	1.465 (2)	C13—H13A	0.9600
C1—H1A	0.9600	C13—H13B	0.9600
C1—H1B	0.9600	C13—H13C	0.9600
C1—H1C	0.9600	C14—C18	1.378 (2)
C1—H1C1	0.9599	C15—C16	1.356 (2)
C1—H1C2	0.9600	C15—H15	0.9300
C1—H1C3	0.9600	C16—C17	1.369 (3)
C3—H3A	0.9600	C16—H16	0.9300
C3—H3B	0.9600	C17—C18	1.371 (3)
C3—H3C	0.9600	C17—H17	0.9300
C4—C5	1.400 (2)	C18—H18	0.9300
C5—C6	1.418 (2)		
			
C2—N1—C6	124.79 (12)	C8—C7—C5	124.30 (13)
C2—N1—C1	117.35 (13)	C9—C8—C7	123.72 (14)
C6—N1—C1	117.85 (13)	C9—C8—N3	114.66 (13)
C2—N2—C4	123.63 (13)	C7—C8—N3	121.48 (13)
C2—N2—C3	117.81 (13)	C10—C9—C8	117.86 (14)
C4—N2—C3	118.32 (13)	C10—C9—H9	121.1
O4—N3—O5	124.05 (15)	C8—C9—H9	121.1
O4—N3—C8	118.52 (13)	C9—C10—C11	121.88 (13)
O5—N3—C8	117.33 (15)	C9—C10—N4	118.44 (15)
O6—N4—O7	123.52 (16)	C11—C10—N4	119.67 (16)
O6—N4—C10	118.22 (18)	C12—C11—C10	118.68 (15)
O7—N4—C10	118.26 (17)	C12—C11—H11	120.7
N1—C1—H1A	109.5	C10—C11—H11	120.7
N1—C1—H1B	109.5	C11—C12—C7	123.00 (14)
N1—C1—H1C	109.5	C11—C12—H12	118.5
N1—C1—H1C1	109.5	C7—C12—H12	118.5
N1—C1—H1C2	109.5	C14—N5—C15	123.68 (13)
H1C1—C1—H1C2	109.5	C14—N5—H5A	118.2
N1—C1—H1C3	109.5	C15—N5—H5A	118.2
H1C1—C1—H1C3	109.5	C14—C13—H13A	109.5
H1C2—C1—H1C3	109.5	C14—C13—H13B	109.5
O1—C2—N1	122.01 (14)	H13A—C13—H13B	109.5
O1—C2—N2	121.56 (15)	C14—C13—H13C	109.5
N1—C2—N2	116.43 (12)	H13A—C13—H13C	109.5
N2—C3—H3A	109.5	H13B—C13—H13C	109.5
N2—C3—H3B	109.5	N5—C14—C18	117.29 (15)
H3A—C3—H3B	109.5	N5—C14—C13	117.48 (13)
N2—C3—H3C	109.5	C18—C14—C13	125.23 (15)
H3A—C3—H3C	109.5	N5—C15—C16	119.76 (16)
H3B—C3—H3C	109.5	N5—C15—H15	120.1
O3—C4—N2	116.74 (13)	C16—C15—H15	120.1
O3—C4—C5	125.13 (13)	C15—C16—C17	118.83 (17)
N2—C4—C5	118.13 (13)	C15—C16—H16	120.6
C4—C5—C6	120.76 (12)	C17—C16—H16	120.6
C4—C5—C7	119.19 (12)	C16—C17—C18	120.25 (16)
C6—C5—C7	119.92 (12)	C16—C17—H17	119.9
O2—C6—N1	117.79 (13)	C18—C17—H17	119.9
O2—C6—C5	126.04 (13)	C17—C18—C14	120.18 (17)
N1—C6—C5	116.17 (13)	C17—C18—H18	119.9
C12—C7—C8	114.74 (13)	C14—C18—H18	119.9
C12—C7—C5	120.86 (13)		
			
C6—N1—C2—O1	−177.51 (15)	C5—C7—C8—C9	172.31 (14)
C1—N1—C2—O1	1.1 (2)	C12—C7—C8—N3	171.51 (13)
C6—N1—C2—N2	2.3 (2)	C5—C7—C8—N3	−12.2 (2)
C1—N1—C2—N2	−179.10 (15)	O4—N3—C8—C9	134.74 (15)
C4—N2—C2—O1	178.35 (15)	O5—N3—C8—C9	−41.7 (2)
C3—N2—C2—O1	4.1 (2)	O4—N3—C8—C7	−41.1 (2)
C4—N2—C2—N1	−1.5 (2)	O5—N3—C8—C7	142.42 (15)
C3—N2—C2—N1	−175.71 (15)	C7—C8—C9—C10	2.6 (2)
C2—N2—C4—O3	−177.37 (14)	N3—C8—C9—C10	−173.16 (14)
C3—N2—C4—O3	−3.1 (2)	C8—C9—C10—C11	0.6 (2)
C2—N2—C4—C5	1.8 (2)	C8—C9—C10—N4	−178.24 (14)
C3—N2—C4—C5	176.00 (15)	O6—N4—C10—C9	−0.4 (2)
O3—C4—C5—C6	176.28 (14)	O7—N4—C10—C9	179.87 (15)
N2—C4—C5—C6	−2.8 (2)	O6—N4—C10—C11	−179.24 (17)
O3—C4—C5—C7	0.5 (2)	O7—N4—C10—C11	1.0 (2)
N2—C4—C5—C7	−178.55 (12)	C9—C10—C11—C12	−2.1 (2)
C2—N1—C6—O2	177.41 (14)	N4—C10—C11—C12	176.75 (14)
C1—N1—C6—O2	−1.2 (2)	C10—C11—C12—C7	0.5 (2)
C2—N1—C6—C5	−3.3 (2)	C8—C7—C12—C11	2.4 (2)
C1—N1—C6—C5	178.11 (14)	C5—C7—C12—C11	−174.04 (13)
C4—C5—C6—O2	−177.32 (14)	C15—N5—C14—C18	1.4 (2)
C7—C5—C6—O2	−1.6 (2)	C15—N5—C14—C13	−177.90 (16)
C4—C5—C6—N1	3.4 (2)	C14—N5—C15—C16	−0.7 (2)
C7—C5—C6—N1	179.18 (12)	N5—C15—C16—C17	−0.6 (3)
C4—C5—C7—C12	133.84 (15)	C15—C16—C17—C18	1.1 (3)
C6—C5—C7—C12	−41.96 (19)	C16—C17—C18—C14	−0.4 (3)
C4—C5—C7—C8	−42.2 (2)	N5—C14—C18—C17	−0.8 (3)
C6—C5—C7—C8	142.00 (14)	C13—C14—C18—C17	178.41 (18)
C12—C7—C8—C9	−3.9 (2)		

### Hydrogen-bond geometry (Å, º)

**Table:**

*D*—H···*A*	*D*—H	H···*A*	*D*···*A*	*D*—H···*A*
N5—H5*A*···O3	0.86	1.82	2.6645 (16)	168
C13—H13*B*···O5^i^	0.96	2.42	3.340 (2)	161
C13—H13*C*···O1^ii^	0.96	2.42	3.160 (2)	134
C15—H15···O2^iii^	0.93	2.29	3.021 (2)	135
C16—H16···O6^iv^	0.93	2.58	3.323 (2)	138
C17—H17···O1^v^	0.93	2.52	3.303 (2)	143

Symmetry codes: (i) −*x*+1, −*y*+1, −*z*+1; (ii) −*x*+1/2, *y*−1/2, −*z*+1/2; (iii) −*x*+3/2, *y*−1/2, −*z*+1/2; (iv) −*x*+2, −*y*+1, −*z*+1; (v) *x*+1/2, −*y*+1/2, *z*+1/2.
